# Behavior of Phenols and Phenoxyacids on a Bisphenol-A Imprinted Polymer. Application for Selective Solid-Phase Extraction from Water and Urine Samples

**DOI:** 10.3390/ijms12053322

**Published:** 2011-05-20

**Authors:** Eliseo Herrero-Hernández, Rita Carabias-Martínez, Encarnacion Rodríguez-Gonzalo

**Affiliations:** 1 Institute of Natural Resources and Agrobiology (IRNASA-CSIC), 37008 Salamanca, Spain; E-Mail: eliseo.herrero@irnasa.csic.es; 2 Department of Analytical Chemistry, Nutrition and Food Sciences, University of Salamanca, 37008 Salamanca, Spain; E-Mail: rcm@usal.es

**Keywords:** molecularly imprinted polymer, precipitation polymerisation, Scatchard analysis, bisphenol-A, phenolic compounds, phenoxyacid herbicides

## Abstract

A molecularly imprinted polymer (MIP), obtained by precipitation polymerisation with 4-vinylpyridine as the functional monomer, ethylene glycol dimethacrylate as cross-linker, and bisphenol-A (BPA) as template, was prepared. The binding site configuration of the BPA-MIP was examined using Scatchard analysis. Moreover, the behaviour of the BPA-MIP for the extraction of several phenolic compounds (bisphenol-A, bisphenol-F, 4-nitrophenol, 3-methyl-4-nitrophenol) and phenoxyacid herbicides such as 2,4-D, 2,4,5-T and 2,4,5-TP has been studied in organic and aqueous media in the presence of other pesticides in common use. It was possible to carry out the selective preconcentration of the target analytes from the organic medium with recoveries of higher than 70%. In an aqueous medium, hydrophobic interactions were found to exert a remarkably non-specific contribution to the overall binding process. Several parameters affecting the extraction efficiency of the BPA-MIP were evaluated to achieve the selective preconcentration of phenols and phenoxyacids from aqueous samples. The possibility of using the BPA-MIP as a selective sorbent to preconcentrate these compounds from other samples such as urine and river water was also explored.

## 1. Introduction

Phenolic compounds are environmental pollutants whose sources may be as different as industrial effluents or the reaction products and degradation of certain pesticides, *etc*. Bisphenol-A (BPA) and -F (BPF) are widely used for the production of epoxy resins and polycarbonate plastics and they can be released into the environment both directly and indirectly. In recent years, special interest has been focused on BPA since *in vitro* experiments have shown it to have estrogenic activity, even at low concentration levels [1]. Phenoxyacetic acids are a class of important plant growth regulators and herbicides that are widely used in agriculture.

MIPs are synthetic materials possessing specific cavities specially designed for the recognition of a given analyte or a group of structurally related species. The synthesis of MIPs involves the assembly of monomers around a template molecule, followed by polymerisation in the presence of a cross-linker. Removal of the template molecule by extraction affords sites specifically available for the insertion of template-like molecules as regards both shape and chemical functionality. Certain qualities of MIPs, such as tolerance to extreme pH values and organic environments, apart from their selectivity, have brought MIPs to the forefront in contemporary chemical research [2].

Depending upon the nature of the chemical bonds involved, MIP synthesis techniques can be classified within three different imprinting approaches: Covalent, semi-covalent and non-covalent. Of these three strategies, the latter has been most widely adopted owing to its experimental simplicity and to the commercial availability of different monomers that are able to interact with almost all kinds of template [3].

Originally, bulk polymerisation was the first strategy used to synthesize imprinted polymers. The monolith is ground and sieved to obtain appropriately sized particles with an irregular shape for further use [4]. The particles thus obtained invariably show a heterogeneous particle size distribution, with poor binding site accessibility for the target analyte. In recent years, considerable efforts have been devoted to developing new polymerisation methodologies for the collection of MIP beads with suitable physical characteristics (size, porosity, pore volume, surface area). Microspheres of regular size and shape can be prepared by precipitation polymerisation, emulsion polymerisation [5], suspension polymerisation [6] and seed polymerisation [7,8]. However, while in the majority of cases stabilizers and surfactants are required for the synthesis procedures, these being additives that may contaminate the final products, precipitation polymerisation has been proposed as a simple and easy strategy for the rapid collection of MIP beads in high yield [9,10]. This method consists of carrying out the polymerisation in a larger amount of porogen than that typically used in the bulk method (2 to 10 times higher). As the polymerisation proceeds, the growing polymer chains become insoluble in the liquid phase and they precipitate. A modified precipitation polymerization method has been reported which results in more uniformly sized microspheres, specially indicated for utilization as selective stationary phase in chromatography [11].

To date, molecularly imprinted polymers (MIPs) have been extensively exploited in many different applications, including their use as separation materials [12,13], chemical sensors [14], reaction catalysts [15] and, in particular, as solid-phase extraction (SPE) sorbents. The introduction of MIPs into SPE, a technique commonly referred to as MISPE, is emerging as a very popular tool. In recent years several publications have reported the success of MISPE in the extraction of a broad range of compounds from matrices as different as water [16,17], honey [18], wine [19], hair [20], urine [21] and cheese products [22], among others.

Recently, different publications addressing the development of BPA-imprinted polymers [23,24], even combined with sol-gel technology [25], have appeared. However, only a few authors have reported their applications as SPE sorbents [26–28] or fibre coatings for SPME [29]. Likewise, MIPs for phenoxyacetic acid herbicides have proliferated [30], and SPE cartridges have been explored with a view to finding suitable applications for the clean-up and pre-concentration of aqueous samples containing chlorinated phenoxyacids [31].

In the present work we used a BPA-MIP, prepared by precipitation polymerization using 4-vinylpyridine (4-VP) as the functional monomer and ethylene dimethacrylate (EDMA) as the cross-linker, as a molecularly imprinted solid-phase extraction sorbent. Our aim was to understand the origin of its recognition properties and to be able to evaluate the parameters that are important in determining the ability of MIPs to recognize template molecules. Moreover, the number of binding sites of the MIP was examined using Scatchard analysis. In addition, the binding selectivity of MIPs and their subsequent recognition mechanism in organic and aqueous media were also explored in detail. The possibilities offered by the BPA-MIP studied here in the direct extraction of these compounds from aqueous samples were also studied. Another objective of our work was to explore the possible applications of the imprinted polymer for the selective, efficient and fast solid-phase extraction of the above-mentioned compounds from complex samples such as urine and river water.

## 2. Results and Discussion

**Figure 1.**
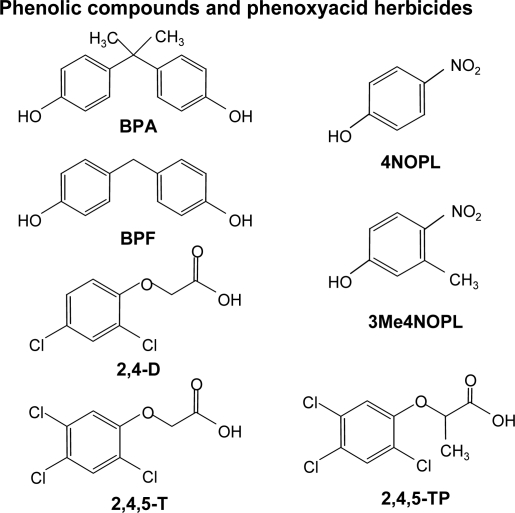

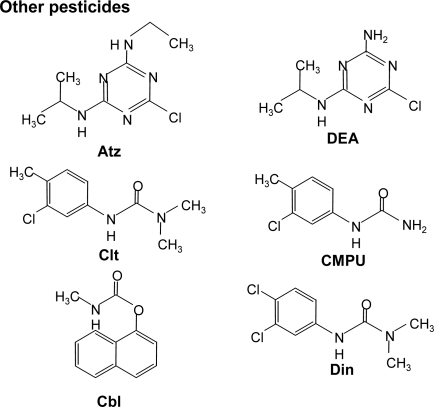
Chemical structures of all the compounds studied.

An imprinted polymer was prepared by precipitation polymerization using BPA as a template and 4-vinylpyridine as the functional monomer, together with a corresponding non-imprinted polymer. This polymer has been developed for use as a sorbent for solid-phase extraction for the preconcentration of phenolic compounds and phenoxyacid herbicides in the presence of other xenobiotic compounds. The chemical structures of the compounds studied are shown in Figure 1.

**Figure 2.**
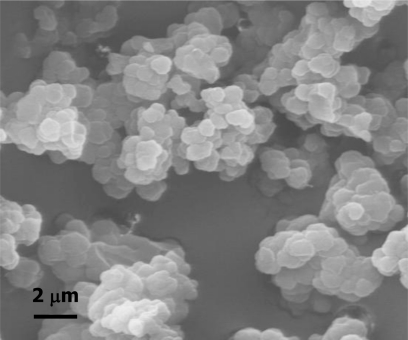
Scanning electron micrograph of the polymer obtained by precipitation polymerisation.

To visualize particle morphologies, scanning electron microscopy (SEM) micrographs were taken of the samples after the template molecule had been extracted. Figure 2 shows a micrograph of the imprinted polymer. As result of the precipitation polymerisation, agglomerates of small spheres with a uniform particle diameter of approx 0.8 μm were obtained.

### 2.1. Binding Performance of the MIP

**Figure 3.**
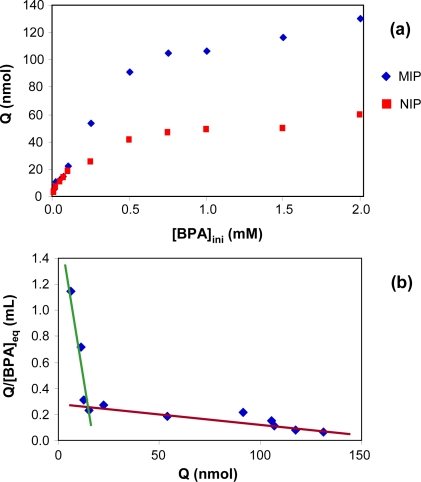
(**a**) Isotherms of the binding of BPA to the imprinted and non-imprinted polymers. Weight of polymer: 10 mg; volume of bisphenol-A standard solution in toluene: 1mL; binding time: 3 h; (**b**) Scatchard plot analysis of the binding of BPA to the imprinted polymer. *Q* is the amount of BPA bound to the MIP. [BPA]_eq_ is the concentration of free bisphenol-A at equilibrium.

To estimate the binding affinity of the MIP for bisphenol-A in toluene, a saturation binding experiment and Scatchard analysis were carried out. The binding isotherms of bisphenol-A to the MIP and NIP were measured at several concentrations in the 0.01–2 mM range (Figure 3a). The amount of BPA bound to the MIP at binding equilibrium, Q, increased together with the increase in the initial concentration of BPA ([BPA]_ini_), and reached saturation at a higher concentration. On comparing the curves obtained for the MIP and the NIP, it may be seen that the amount of template bound to the imprinted polymer was much higher than that bound to the non-imprinted polymer. This suggests that the imprinted cavities of the MIP may be responsible for the high-affinity binding of the template to the polymer. The binding characteristics of MIPs can be estimated using Scatchard analysis. The Scatchard equation is:

$${Q/\left[\text{BPA}\right]}_{\text{eq}}=\left(Q_{\text{max}}-Q\right)/K_{\text{D}}$$

where *Q* is the amount of bisphenol-A bound to the MIP at equilibrium; *Q*_max_ is the apparent maximum number of binding sites; [BPA] is the free BPA concentration at equilibrium, and *K*_D_ is the equilibrium dissociation constant of the binding sites. *Q*/[BPA] was plotted *vs. Q*, as shown in Figure 3b. The Scatchard plot for the MIP is not a single linear curve: there are two distinct sections within the plot, with different slopes. This suggests that there are two classes of heterogeneous binding sites as regards affinity for bisphenol-A in the polymer. The linear regression equation for the left part of the curve in the figure is *Q*/[BPA]_eq_ = −0.0975*Q* + 1.6786; the unit of *Q* is nmol. *K*_D_ and *Q*_max_ were calculated to be 10.3 μmol L^−1^ and 17.3 μmol g^−1^ of dry polymer, respectively, from the slope and the intercept of the Scatchard plot. The linear regression equation for the right part of this curve is *Q/*[BPA]_eq_ = −0.0016*Q* + 0.2806. *K*_D_ and *Q*_max_ were calculated to be 625.0 μmol L^−1^ and 175.4 μmol g^−1^ of dry polymer.

It may be concluded that the binding site configuration of the MIP is heterogeneous as regards affinity for bisphenol A, and this indicates that the binding sites can be classified within two groups with different binding properties.

A similar experiment was carried out in aqueous medium, but in this case nearly all the BPA was retained in the MIP and the NIP, regardless of the concentration.

### 2.2. Study of the Retention Process in Organic Medium

The purpose of the present work was to develop a novel MIP for the extraction of certain phenolic compounds from different matrices. In this sense, the sorbent used should ideally be compatible with both organic and aqueous media. Thus, the extraction column can operate either in the “selective adsorption” mode, for which the sample matrix containing the analyte can be dissolved or extracted with an organic solvent, or in the “selective desorption” mode, which is especially suitable for aqueous samples.

In light of the different selective retention mechanisms in which MIPs can operate, we initially studied the ability of the MIP obtained by precipitation polymerization to specifically recognize analytes from organic medium. As is known, MIPs often exhibit a stronger imprinting effect in the solvent in which they were originally obtained (usually toluene). This was evaluated by working in parallel with cartridges filled with imprinted polymer (MIP) and non-imprinted polymer (NIP), both obtained as described in the experimental section. The analytes included in this study belong to different families and can be classified in the following subgroups: phenolic compounds (BPA, BPF, 4-NOPL, 3-Me4-NOPL), phenoxyacid herbicides (2,4-D; 2,4,5-T and 2,4,5-TP), and other pesticides and some of their degradation products (atrazine and its metabolite DEA, chlortoluron and its metabolite CMPU, diuron and carbaryl).

**Figure 4.**
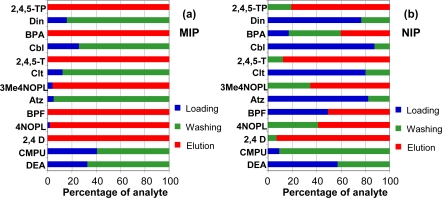
Percentage of each of the compounds studied in the loading, washing and elution steps of the (**a**) MIP and (**b**) NIP. Sample: 5 mL of toluene spiked at a concentration of 100 μg L^−1^. Washing step: 10 mL of dichloromethane. Elution step: 10 mL of acetonitrile:acetic acid (9:1, v/v).

The loading step was accomplished using 5 mL of a standard mixture in toluene spiked with all the analytes studied at a concentration of 100 μg L^−1^. In order to prevent non-specific interactions between the analytes and the MIP, a cartridge-washing step was implemented, initially using 10 mL of dichloromethane, because this was the solvent that had produced the best results in a previous work with a propazine-imprinted polymer [32]. All the fractions obtained in the loading, washing, and elution steps were collected, evaporated and analyzed. The results obtained (Figure 4) show that the phenols and phenoxyacid herbicides were completely retained in the MIP (Figure 4a). In contrast, in the case of the NIP (Figure 4b) the phenoxyacid herbicides were retained, with recoveries higher than 80%, but the phenols had lower recovery values. This behaviour shows that the retention of phenolic and phenoxyacid compounds in organic medium is due to more than one type of interaction. The rest of the compounds (DEA, CMPU, Atz, Clt, Cbl and Din) were mainly eliminated in the washing step, in the case of the MIP, or were not retained, in the case of the NIP. Accordingly, the results obtained indicate that toluene is a suitable organic medium for the retention of phenols and phenoxyacid herbicides by the BPA-MIP.

#### 

##### Influence of the Amount of Sorbent Used

**Figure 5.**
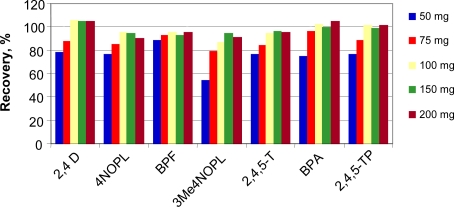
Influence of polymer mass on the recoveries of the fraction eluted from the bisphenol-A-MIP. Sample: 5 mL of toluene with 100 μg L^−1^ of each compound. Washing and elution steps as in Figure 4.

The amount of polymer to be used is an important issue to be considered when employing a solid sorbent. To check the influence of this in the recovery values, empty SPE cartridges were filled with different amounts of polymer. For most of the compounds, the recoveries obtained were not significantly better when amounts of sorbent above 100 mg were used. Bearing in mind that the difficulty in passing the sample through the system increases with the increase in polymer mass, we decided to use 100 mg of polymer as a compromise amount (Figure 5).

### 2.3. Study of the Process of Retention in Aqueous Medium

With a view to being able to use the MIP for the direct solid-phase extraction of xenobiotic compounds from several aqueous matrices, the behaviour of this imprinted polymer as a SPE sorbent was studied.

The BPA-MIP was exposed to a standard aqueous solution (5 mL) containing all the xenobiotic compounds (100 μg L^−1^). The recoveries obtained when the imprinted and non-imprinted polymers were used as sorbents are shown in Table 1, where it may be seen that the values for the imprinted polymer are higher than 75% for both the phenolic compounds and the phenoxyacid herbicides. These compounds were also retained to a slight extent in the NIP, but in this case recoveries were lower than 30%, except for BPF, for which the recovery was 49%. The other compounds were not retained either in the MIP or in the NIP. This suggests that the BPA-MIP can be used to selectively preconcentrate phenols and phenoxyacid herbicides from aqueous samples.

#### 2.3.1. Influence of the Washing Solvent

The choice of washing solvent is an important factor when desiring to increase selectivity. In order to obtain the best results, three solvents were assayed: toluene, dichloromethane and acetonitrile. Optimum results were obtained with dichloromethane; the recoveries obtained with acetonitrile were lower than those obtained with toluene and dichloromethane, and selectivity was higher with dichloromethane.

Once it had been decided that dichloromethane was the best washing solvent, the influence of the volume of this solvent was assessed. To accomplish this, the MIP was washed successively with increasing volumes of dichloromethane and the fractions thus obtained were analysed chromatographically (Table 2). It was observed that the compounds of the different families of the phenols and phenoxyacid herbicides were eluted efficiently (>80%) in the first two fractions of dichloromethane. In contrast, the compounds retained specifically were not eluted in these fractions; BPA only began to be eluted in the third fraction. The phenoxyacid herbicides and the smallest phenolic compounds (4NOPL and 3Me4NOPL) were not eluted, even when 20 mL of dichloromethane was used.

These results prompted us to employ a compromise solution, in which, by using a washing step with 5 mL of dichloromethane, the phenolic compounds and the phenoxyacid herbicides could interact with the MIP in a specific way. The results indicated that, when the sample was passed through the cartridge in aqueous medium, the analytes were retained by non-selective interactions. In contrast, washing later with dichloromethane generated an organic medium in which non-selective interactions were converted into interactions specific for the phenolic and phenoxyacid compounds [32,33].

#### 2.3.2. Effect of Drying Time

The retention of the analytes on a sorbent from an aqueous medium may be strongly affected by the presence of water embedded in the sorbent after sample passage. Accordingly, we next studied how the drying time affected the retention of the analytes in the MIP.

After passing an aqueous sample through the system, the sorbent was dried by applying a vacuum of −15 mmHg for different times (5, 30 and 60 min). Following this, it was also dried in an oven at 50 °C for 12 h. The recoveries obtained for drying times of 5, 30 and 60 min were similar. By contrast, when the sorbent was dried in an oven at 50 °C for 12 h, a decrease in the recoveries obtained for the phenolic compounds was observed, probably due to their volatility.

#### 2.3.3. Influence of the Volume of the Elution Solvent

The elution of the compounds retained specifically was conducted with a acetonitrile:acetic acid mixture (9:1 v/v), although considering that after the elution it is necessary to include an evaporation step, the minimum solvent volume required for elution was studied. To accomplish this, the MIP was loaded with 10 mL of UHQ water spiked with 50 μg L^−1^ of each analyte, washed with 5 mL of dichloromethane, and successively eluted with increasing volumes of the acetonitrile:acetic acid mixture. The fractions thus obtained were analysed chromatographically. It was observed that the first 2.5-mL fraction of the acetonitrile:acetic acid mixture eluted the highest amounts, and for more polar compounds elution was even complete. The second 2.5-mL fraction eluted small amounts of 2,4,5-T, BPA and 2,4,5-TP. None of the analytes were detected in successive fractions. A volume of 5 mL of acetonitrile-acetic acid (9:1 v/v) was therefore used as the elution solvent in later studies.

#### 2.3.4. Effect of Sample pH on Recovery Efficiency

The retention of analytes on the structure of MIPs may be influenced by pH, and this can may significantly different between several water sources. Some authors [33] have pointed out that a low pH level is optimal for the retention of acidic phenolic compounds when an MIP is synthesized using 4-VP as the functional monomer. Since this was the functional monomer used here to obtain the MIP, a study of the influence of sample pH on recoveries was performed. To accomplish this, samples of bottled water were loaded in the MIP at three different pH levels (2.9, 6.0 and 9.3, adjusted with ammonium formate buffer or boric-borate buffer). The results showed that pH did not affect the recoveries to a significant extent, and no general trend could be established. This kind of behaviour can be explained considering that retention takes place through hydrophobic interactions and electrostatic interactions. The analytes studied here would ionize at neutral or alkaline pH, and this ionization would favour the development of electrostatic interactions. In an acid medium, even if the analytes were not ionized, the functional monomer would ionize and, again, electrostatic interactions could be established [33,34].

### 2.4. Use of the BPA-MIP as Selective Sorbent from Water and Urine Samples

Apart from the characterisation of the binding sites and the selectivity study described above, another objective of this work was to explore the possible applications of the imprinted polymer for the selective extraction of the xenobiotic compounds studied from real samples.

**Figure 6.**
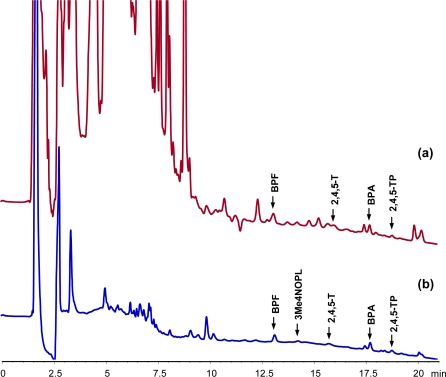
Chromatograms obtained for the MISPE extraction of 20 mL urine spiked with 25 μg L^−1^ of each analyte, without (**a**) and with (**b**) the addition of 5% of acetonitrile to the sample. Washing step: 5 mL of dichloromethane. Elution step: 5 mL of acetonitrile:acetic acid (9:1, v/v).

Urine is representative of highly complex matrices. Figure 6a shows the chromatogram obtained when 20 mL of urine (from healthy volunteers, and previously frozen, thawed and filtered) spiked with all the analytes (phenols, phenoxyacids, DEA, CMPU, Atz, Clt, Cbl and Din) at 25 μg L^−1^ was passed through the MIP and analyzed following the indicated procedure. Peaks corresponding to BPF, 2,4,5-T, BPA and 2,4,5-TP were detected. Also, a broad band appeared at the beginning of the chromatogram. In order to obtain a cleaner chromatogram, an additional step was included in the MISPE protocol; this involved the addition of 5% (v/v) of acetonitrile to the urine sample before passing it through the MIP. The introduction of this new step in the extraction procedure produced cleaner chromatograms (Figure 6b), and peaks corresponding to BPF, 3Me4NOPL, 2,4,5-T, BPA and 2,4,5-TP were readily identifiable and the recovery values were in the range 54% for 3Me4NOPL and 96% for BPA, with detection limits, for a signal to noise ratio of 3, in the range 5.3 μg L^−1^ for 3Me4NOPL and 0.9 μg L^−1^ for BPA and 2,4,5-TP. Thus, the MISPE procedure proved to be simple and effective for the elimination of substances that can interfere in analyte detection or that may damage the chromatographic system. To improve the detection limits in urine samples, the appropriate sample treatment should be optimized.

**Figure 7.**
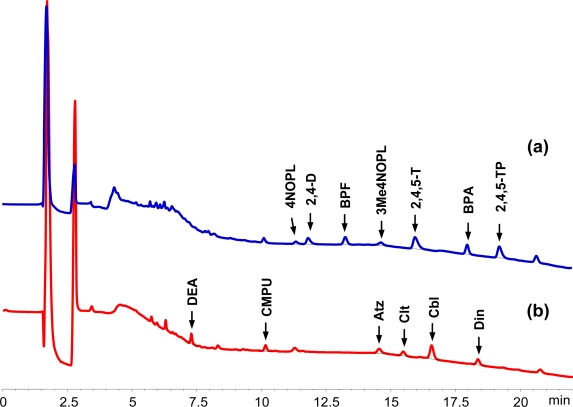
Chromatograms obtained for the MISPE extraction of 100 mL of Tormes river water spiked at a level of 0.5 μg L^−1^ corresponding to the elution step (**a**) and the washing step (**b**). Washing and elution steps as in Figure 6.

Water samples from the River Tormes (Salamanca, Spain) were also selected to demonstrate that the BPA-MIP is able to selectively bind these xenobiotic compounds from environmental aqueous matrices. Figure 7 shows the chromatograms corresponding to the washing and elution steps obtained after MISPE treatment of 100 mL of river water spiked with all the analytes studied at a level of 0.5 μg L^−1^. As expected, the band corresponding to humic acids was reduced to a considerable extent, only signals that correspond to phenols and phenoxyacid herbicides are present (Figure 7a) and its identification and quantification is easier. The washing step with dichloromethane removes all the non-retained compounds, allowing even the identification and quantification of these compounds (Figure 7b). Table 3 shows the recoveries and the limits of detection for the compounds retained in the MIP (detected in the elution step) and also the data corresponding to the non-retained compounds (detected in the washing step with dichloromethane). As can be seen, recoveries obtained were >75% for all the retained compounds; even in the case of non-retained compounds (other pesticides and metabolites detected in the washing step), the recoveries where high enough to allow quantification. The detection limits were lower than 0.5 μg L^−1^, that is the level of quantification recommended within Europe for the determination of herbicides in surface waters.

## 3. Experimental Section

### 3.1. Chemicals

The xenobiotic compounds were obtained from Sigma-Aldrich (Steinheim, Germany) and were used without further purification. The compounds studied were as follows: *bisphenol-A* (BPA), 2,2-bis(4-hydroxyphenyl)propane, CAS RN [80-05-7]; *bisphenol-F* (BPF), bis-(4-hydroxyphenyl)methane, CAS RN [620-92-8]; *2,4-dichlorophenoxyacetic acid* (2,4-D), CAS RN [94-75-7]; *2,4,5-trichlorophenoxyacetic acid* (2,4,5-T), CAS RN [93-76-5]; *2-(2,4,5-trichlorophenoxy)propionic acid* (2,4,5-TP), CAS RN [93-72-1]; *4-nitrophenol* (4NOPL), *p*-nitrophenol CAS RN [100-02-7]; 3-methyl-4-nitrophenol (3Me4NOPL), 4-nitro-*m*-cresol CAS RN [2581-34-2]. Stock solutions of each analyte were prepared in acetonitrile at 500 μg mL^−1^.

Other pesticides studied were obtained from Ehrenstorfer (Augsburg, Germany): *atrazine* (Atz), 6-chloro-*N*^2^-ethyl-*N*^4^-isopropyl-1,3,5-triazine-2,4-diamine CAS RN [1912-24-9]; *chlortoluron* (Clt), 3-(3-chloro-*p*-tolyl)-1,1-dimethylurea CAS RN [15545-48-9]; *CMPU* (CMPU), *N*-(3-chloro-4-methylphenyl)urea, CAS RN [590393-14-9]; *carbaryl* (Cbl), 1-naphthyl-*N*-methylcarbamate CAS RN [63-25-2] and *diuron* (Din), 3-(3,4-dichlorophenyl)-1,1-dimethylurea CAS RN [330-54-1].

4-Vinyl pyridine (4-VP) and ethylene dimethacrylate (EGDMA) were obtained from Sigma-Aldrich (Steinheim, Germany); 2,2′-azobis(2-methyl-propionitrile) (AIBN) was obtained from Acros organics (Geel, Belgium).

The organic solvents, acetonitrile and methanol were of HPLC grade (Merck, Darmstadt, Germany) and were used as received. Dichloromethane and acetic acid were of analysis grade (Scharlau, Barcelona, Spain).Ultra-high quality (UHQ) water was obtained with an Elgastat UHQ water purification system.

All other chemicals were of analytical reagent grade.

### 3.2. Synthesis and Characterisation of the Molecularly Imprinted Polymer

The procedure followed to obtain the MIP by precipitation polymerisation has been described before by the authors [17,18]. The morphology of the polymer was determined by scanning electron microscopy, using a Zeiss DSM 949 (Zeiss, Oberkochen, Germany) device at the Electron Microscopy Service of the University of Salamanca.

Recovery values (mean of three experiments) were determined by relating the signal of the analytes eluted with a mixture of acetonitrile:acetic acid (9:1, v/v) from the corresponding polymers with the concentration to be expected if recoveries were 100%.

### 3.3. Binding Experiments

A series of BPA standard solutions was prepared in toluene. One-millilitre aliquots of each solution were mixed with 10 mg of imprinted polymer and non-imprinted polymer particles in a 2 mL glass vial. The vials were shaken in a constant-temperature bath at room temperature for 3 h. After the binding process was completed, the mixture was filtered with a 0.45-μm filter. The BPA concentration in the filtrate was measured by HPLC-DAD UV and the amount of BPA bound to the MIP and NIP was calculated by subtracting the concentration of free BPA from the initial concentration.

### 3.4. MISPE Procedure in Organic Medium

100-mg samples of each polymer were dry-packed in empty solid-phase extraction cartridges and conditioned with 5.0 mL of an acetonitrile:acetic acid mixture (9:1, v/v), 5.0 mL of dichloromethane, and 5.0 mL of toluene. For the experiments involving extraction from organic samples, the MIP columns were loaded with 5.0 mL of a mixture of the analytes at a concentration of 100 μg L^−1^ in toluene. After an exhaustive drying step, 10 mL of dichloromethane was percolated through the sorbent to eliminate non-specific interactions. The target analytes were eluted from the cartridge with 5 mL of an acetonitrile:acetic acid (9:1, v/v) mixture. In order to evaluate the efficacy of the polymer, the fractions eluted were collected and evaporated to dryness under a stream of nitrogen. The residues were reconstituted in 1.0 mL of water-acetonitrile (9:1, v/v) and analyzed using an HPLC-DAD UV system.

### 3.5. MISPE Procedure in Aqueous Medium

A procedure similar to that described in the previous section was used for the extraction of the analytes from aqueous samples, the only difference being that in the final conditioning step the solvent employed was water instead of toluene. A volume of 5.0 mL of aqueous sample was passed through the MIP, after which an exhaustive drying step was implemented. The washing and elution steps were performed under the same conditions as those described in the previous section.

### 3.6. Chromatographic Conditions

HPLC-DAD UV was performed on a HP 1100 Series chromatograph from Agilent (Waldbronn, Germany) equipped with a binary pump, a membrane degasser, an autosampler, and a UV diode-array detector (UV-DAD). The system was controlled by an HP ChemStation, which also collected the data from the diode array detector and performed quantitative measurements. The analytical column used was a 150 × 4.60 mm Luna PFP(2) packed with 3 μm particles (Phenomenex, Torrance, CA, USA). The diode array detector was set at 300 nm for 4NOPL and 3Me4NOPL, 244 nm for CMPU, Clt and Din, and 214 nm for all other analytes. Spectra were recorded in the 190–400 nm range.

The mobile phase consisted of an acetonitrile (solvent A)-5 mM ammonium formate buffer, pH = 3.5, (solvent B) isocratic mixture (15:85, v:v) for 1 min; a linear gradient from 15% to 35% of solvent A in 3 min; another isocratic period of 7 min; another linear gradient from 35% to 55% in 13 min, and then a return to the initial conditions in 2 min, with 3 min for equilibrating the column. Flow rate was 1 mL min^−1^ and the volume injected was 100 μL. The analytical column was thermostatted at 25 °C.

### 3.7. Water and Urine Samples

Samples of river water were taken from the River Tormes in the city of Salamanca (Spain). They were collected directly in 1 L glass bottles. All samples were filtered through 0.45 μm pore size cellulosic membrane filters (Osmonics^®^, Kent, WA, USA) and were stored at 4 °C in the dark until extraction. First, all water samples were analyzed using the proposed method to check for the presence of the analytes. No signals corresponding to target analytes were found, and hence spiked water samples were used.

Urine samples collected from four healthy volunteers were used for developing the method and the preparation of calibration standards. Urine samples were collected in 250 mL brown glass bottles and frozen immediately until analysis. Before use, the urine samples were thawed at room temperature and spiked daily with diluted standard solutions. Samples were filtered through 0.45 μm Cameo filters to remove precipitated proteins and 5% (v/v) of acetonitrile was added to the urine sample before passing it through the MIP. All urine samples were first analyzed with the proposed method in order to check the natural occurrence of the target compounds; no signal corresponding to the target analytes were found.

### 3.8. SPE Procedure

Preconcentration was accomplished by passing 100 mL of natural water or 20 mL of urine through an empty extraction cartridge loaded with 100 mg of the BPA-MIP, previously conditioned with 5 mL of dichloromethane, 5 mL of acetonitrile/acetic acid (9:1, v/v) and 10 mL of water. Following this, the cartridges were dried for 30 min under a vacuum of −15 mmHg and then 5 mL of dichloromethane and 5 mL of acetonitrile/acetic acid (9:1, v/v) were used as washing and elution solvents. The fraction eluted was evaporated to dryness and the dry residues were reconstructed in 500 μL of a water/methanol mixture (1:1, v/v).

## 4. Conclusions

In this work, a new molecularly imprinted polymer has been synthesized by precipitation polymerization with bisphenol-A as template and 4-vinylpyridine as the functional monomer. The Scatchard method revealed that in toluene the binding site configuration is heterogeneous as regards the affinity for bisphenol-A. *K*_D_ and *Q*_max_ values of 10.3 μmol L^−1^, 17.3 μmol g^−1^ and 625.0 μmol L^−1^ and 175.4 μmol g^−1^ were calculated, indicating that there are two classes of binding sites in the MIP. The BPA-MIP showed excellent molecular recognition abilities, and not only for phenolic compounds; indeed, even phenoxyacid herbicides were selectively retained in both organic and aqueous media.

The results indicated that careful choice of the washing and elution conditions afforded a reliable MISPE method, and its application in complex samples has been demonstrated. In the analysis of urine samples, it was necessary to add 5% of acetonitrile to the sample before passing it through the MIP. Under these conditions, cleaner chromatograms were obtained and the peaks corresponding to BPF, 3Me4NOPL, 2,4,5-T, BPA and 2,4,5-TP were easily identified. In river water analysis, no relevant pre-treatment of the sample was necessary to obtain clean chromatograms with detection limits lower than 0.5 μg^−1^, that is the level of quantification recommended within Europe for the determination of herbicides in surface waters.

## Figures and Tables

**Table 1.** Recoveries (%) of some xenobiotic compounds employed to study the selectivity of MIP and NIP. Sample: 5 mL of UHQ water spiked with 100 μg L^−1^ of each analyte. Washing solvent: 10 mL of dichloromethane.

**Compound**	**Recoveries (%) ± S.D.^a^**	
	**MIP**	**NIP**
**DEA**	−	−
**CMPU**	6 ± 1	8 ± 1
**4NOPL**	79 ± 8	14 ± 2
**2,4-D**	86 ± 9	16 ± 2
**BPF**	88 ± 8	49 ± 5
**Atz**	−	−
**3Me4NOPL**	75 ± 8	24 ± 3
**Clt**	−	−
**2,4,5-T**	93 ± 8	36 ± 4
**Cbl**	−	−
**BPA**	76 ± 8	22 ± 3
**Din**	−	−
**2,4,5-TP**	84 ± 7	13 ± 2

a S.D. = standard deviation for *n* = 3.

− Not retained.

**Table 2.** Influence of the volume of dichloromethane used in the washing step. Sample: 5 mL of UHQ water spiked with 100 μg L^−1^ of each analyte.

	**Percentage of analyte removed in each fraction**					
	**1st fract. 2.5 mL**	**2nd fract. 2.5 mL**	**3rd fract. 5 mL**	**4th fract. 5 mL**	**5th fract. 5 mL**	**Total washed**
Phenolic compounds and phenoxyacid herbicides						
**4NOPL**	–	–	–	–	–	–
**2,4-D**	–	–	–	–	–	–
**BPF**	–	–	–	7	8	15
**3Me4NOPL**	–	–	–	–	–	–
**2,4,5-T**	–	–	–	–	–	–
**BPA**	–	–	6	13	6	25
**2,4,5-TP**	–	–	–	–	–	–
Other pesticides and metabolites						
**DEA**	76	8	4	–	–	88
**CMPU**	49	21	7	5	3	85
**Atz**	75	9	–	–		84
**Clt**	75	7	4	–	–	86
**Cbl**	76	9	3	–		88
**Din**	73	8	3	–	–	84

**Table 3.** Recoveries and limits of detection obtained for all the compounds studied. Sample: 100 mL of river water spiked with 0.5 μg L^−1^ of each analyte.

	**Recoveries (%) L.D.^a^ (μg L**^−^**^1^)**	
Phenolic compounds and phenoxyacid herbicides		
**4NOPL**	77	0.12
**2,4-D**	78	0.08
**BPF**	84	0.09
**3Me4NOPL**	76	0.09
**2,4,5-T**	98	0.04
**BPA**	92	0.06
**2,4,5-TP**	91	0.04

Other pesticides and metabolites ^b^		
**DEA**	70	0.13
**CMPU**	68	0.15
**Atz**	87	0.08
**Clt**	84	0.11
**Cbl**	77	0.01
**Din**	87	0.13

a D.L. = detection limit for a signal-to-noise ratio of 3;

b non-retained compounds detected in the washing step with dichloromethane.
